# Supplementary material for: A preregistered multi-lab replication of Maier et al. (2014, Exp. 4) testing retroactive avoidance
Source: PLoS One. 2020 Aug 31;15(8):e0238373. doi: 10.1371/journal.pone.0238373 (PMC7458331; doi:10.1371/journal.pone.0238373)
Supplement: Attachment — Submitted filename: Response to Reviewers.docx [file pone.0238373.s003.docx]

Dear Dr. Dr. Naudet,

dear Reviewers,

we would like to thank you for your additional comments on our revised manuscript entitled „A Preregistered Multi-Lab Replication of Maier et al. (2014, Exp. 4) Testing Retroactive Avoidance” submitted for publication to PLOS ONE. We tried to carefully respond to every comment you made. In this document you will find our answers to your comments and a description of how we adjusted the text (see **). We also revised some of the figures as you suggested.

Thank you for the open and fair scientific discussion we had and your profound work on this paper.

First I would like to apologize for the time it took me to do this second round of review: this should have been much faster.

**It is fine, thank you for taking this job seriously.

I would like to thank the authors for their clarifications, all the reworking and additions to the manuscript. It makes it clearer where the line between results and interpretation lie.

Updating the code and the data (from this and the previous study) on OSF have also clear added values.

My two main suggestions are:

- display for figure 3 the null distribution on which the statistical inference is performed
- add a readme to the OSF project and ensure that the code runs as intended when downloaded from source

**Ok, good point. We added a “readme” file and added a figure (Figure 4 a-c) that shows the null distributions of all three exploratory analyses and where the human data lie.

***Although inflation bias is not a problem as outlined above, to circumvent the multiple*

*testing approach in the FFT analysis described, we propose a different and more*

*elegant method for comparing the FFT results from the human data with the FFTs from*

*the 10,000 simulated data sets:*

***To test the FFT results from the human data against chance occurrence, all 1,164*

*amplitudes obtained from the FFT of the human data set were added up creating a*

*sum score of the amplitudes obtained from all tested frequencies of this set. In the*

*same way for each of the 10,000 simulations the sum score of amplitudes was*

*computed. The distribution of the sum scores of amplitudes across all simulations now*

*served as the null distribution (see Figure 5). The sum score of amplitudes of the*

*human data set was Sumamp = 34.84. Only 147 of the simulations (1.47%) reached a*

*sum score of 34.84 or higher. We added this new analysis at the end of the*

*“Exploratory analysis” section and replaced the old one.*

***We are not testing every BF obtained in the human data sequential analyses against the corresponding BFs from the simulations. Rather we identify the single highest one and compare it with a distribution of single highest BFs found in the simulation analyses. Thus, no multiple testing is involved here.*

***But in case we missed something here, our theoretical argument against the problem*

*of inflation would apply here as well (see our response above).*

I thank the authors for explaining this more thoroughly. This helped me understand why I got confused and thought there was a multiple comparison problem. The text did indeed mention what null distribution was used to make the statistical inference. However the figure of the previous version of the article showed a 95% confidence interval envelope that a) was unrelated to the inference made in the text, b) would actually suffer from multiple comparison problem if it were used as a way to threshold and detect extreme data.

**Good point, we deleted the confidence interval (see Figure 3).

The new approach suggested by the author is an improvement as it is more direct and the link between the figure and the text is much clearer.

In the same spirit I would suggest that:

the authors make the text and the figure 3 more congruent and add a figure (or maybe an inset to the figure 3) similar figure 5 showing where the maximum BF values of the human data lies when compared to the distribution of maximums obtained from the simulation.

**Done, see our response above and Figure 4a-c.

Also maybe I am still missing something but if only the maximum BF value is taken for the inference how can **other** maximum BF be found in time series as suggested by this sentence:

“Higher BFs were also found with participants numbered 500 to 600 (i.e., 400 in the early stages of the replication study).”

**Good point, the statement does not match the analysis and was therefore deleted from the text.

*The stimuli and mask presentation time are slightly different between the pre-*

*registration and the manuscript. Is this because the refresh rate of the monitor was*

*different?*

***Yes, this was the reason. We are convinced that these adjustments are not*

*responsible for the null effect (and nobody would argue this way).*

Indeed I was not arguing that way. I was more trying to understand where some of the differences were coming from.

Code

But I admit that the lack of a README did not help in figuring out:

- what packages are needed
- where unzipped files should go
- in which order the scripts should be run as there seem to be some dependencies between them.

I suggest that such basic documentation should be provided.

**Done, see the “readme” document.

I was unable to make some of the R code run (which might be partly due to my lack of R knowledge) and in ‘exploratoryanalyses.R’

Error in rbind(deparse.level, ...) :

numbers of columns of arguments do not match

Could the authors make sure that everything runs as intended from simply downloading the OSF project and trying to run it from there? This is usually even better when performed by a colleague “naive” to the study to ensure a bare minimum of portability to make sure the one’s code does not just run on one’s machine.

**OK, we made sure that now everything works. We also made the independent test as suggested and it worked.

On that note, I would like to point to the authors (and the editors) this recent initiative that makes sure that submitted code runs as intended: <https://codecheck.org.uk/>

**Thank you. This is helpful!

I am appending below additional comments and suggestions of my physicist colleague.

Quantum theory

l.74 : « group of theories » should be replaced by « group of interpretations » (which don’t have the level of certainty of a theory)

GQT is an interpretation, even if it has the arrogance to call itself a theory, without any experiments to support its hypotheses. It doesn’t have the consensual character of a theory, it is only supported by a few physicists and only based on interpretation from the quantum model.

**We agree, this is an important point you make. However, a theory does not need any empirical evidence to be called a theory and consensus is not a feature of a theory either. But we understand what the reviewer wants to say here and we changed the corresponding statement as suggested.

OrchOR is a philosophy of mind, also based on interpretation and extrapolation from the quantum model to the mind, without any supporting experiments or direct observations. Thus, at l.76, “theory” should be replaced by “philosophy of mind”.

**OK.

In my opinion, this is not the kind of foundations that one should build assumptions in other fields upon, so I would suggest that you don’t mention quantum mechanics at all. But if you really want to mention it, it should be clear in your formulation that « if these interpretations are true », then there are similarities with your subject of experiment that would indicate that there could possibly be a causal link between the two hypothetical phenomena. In addition, it should also be very clear that these interpretations are (markedly) minor ones and still debated amongst all the others that have been put forward about the quantum model.

Otherwise, there is a high risk that your article will be automatically considered by any physicist who would read it as deliberately deforming the current consensus in physics in order to match its own conclusions, which would be regrettable if physics and psychology are as interdependent as your article would suggest, because in this case this research field could only benefit from collaboration with physicists.

** We hear you on this and added several statements in the text that put the interpretations into the context of all the other interpretations of QM.

**Just for the record: If someone really wants to put forward an innovative idea at some point one has to challenge the current consensus or parts of it, otherwise no progress will ever be made. Whether our approach was valid or not was put to an empirical test and we were found to be wrong. So, the current consensus remains intact. We make this very clear in our paper especially in the discussion section.

I understand better now what you mean by « temporal non-locality » and I was aware of the kind of phenomenon that it stands for in your article (which appears in delayed-choice experiments). The formulation is a little confusing, because « locality » etymologically refers to a « location » (in space) so maybe it would be clearer to call it « non-temporality » (this is not an official denomination).

**According to Einstein’s general relativity theory space and time are inseparable. So, locality as the term is used in general relativity (see also Einstein, Podolsky & Rosen, 1935) always includes both time and space.

I’ve found two or three articles that use the same denomination as you do but a few others use « temporal Bell inequalities » or « temporal entanglement », actually it doesn’t have an official name because it’s only an aspect of the intrication phenomenon in general.

The real experimental effect is called « delayed-choice experiments », but the choice to consider that there is a new physical phenomenon (« temporal non-locality » or else) underlying this effect depends on the interpretation. Notably in the standard interpretation it’s just an effect of observation, and in GQT it will be the same because of the role of consciousness in the observation (basically put : in delayed-choice experiments, there are unobservable events which happen before the observation that are correlated to the choice made by the observer while doing the observation later. But in GQT, only the consciousness is capable of producing a measurement, so nothing unobservable ever happens, so there is no correlation, everything is only determined when the observer arrives).

**Yes, but consciousness can determine later what has happened before and not only what occurs during the observation.

This is the only interpretation of quantum mechanics that you refer to (since OrchOR is not really an interpretation of quantum mechanics but rather an application of it to the philosophy of mind). Be careful then because the effect you refer to with « temporal non-locality » doesn’t exist in the interpretation that you refer to.

**It does exist, both in GQT and OrchOr as confirmed from the authors of GQT in personal communication and with regard to orchOr also explicitly in the paper of Hameroff (2012):

[26] Hameroff S. How quantum brain biology can rescue conscious free will. Frontiers in Integrative Neuroscience. 2012; 6(1): 93. <https://doi.org/10.3389/fnint.2012.00093>

I would suggest that you choose to mention only one of these ideas (non-temporality or GQT) for consistency’s sake.

**See our response above. In addition, we are only referring to what Maier et al.’s arguments were in 2014. Thus, we would like to keep the line of arguments as it is. We already shortened it to a minimum of information.

If you’re looking for an interpretation that would consider the delayed-choice experiments as a manifestation of time bidirectionality, there is the transactional interpretation, but this one doesn’t make any reference to consciousness so it cannot be linked to the rest of your work.

**Thank you for the hint. And yes, we know about TI and there are also attempts to modify it to include consciousness, but we did not want to make the theoretical part bigger (and more speculative) than absolutely needed.

I would consider the reference to non-temporality (or “temporal non-locality”) as the weakest part because if this effect is real it would typically be destroyed very quickly by decoherence (the statistical process that makes quantum effects very unprobable and unstable at large scales and usual temperatures), so it would be very unprobable that it could be maintained through a whole room full of air in order to carry retrocausal information all the way from the stimuli to the subject’s brain.

**Yes, unlikely but not impossible, that is the point. Our kind of work tried to figure out those boundary conditions under which temporal non-locality could be observed macroscopically. Nowadays, superposition states of relatively large objects are found that -given decoherence- would have been considered highly unlikely years ago.

l.84 : « implies » should be replaced by « could be explained by the hypothesis that », because it’s not a necessary implication. As I mentioned, the delayed choice experiments can be interpreted in several ways (I'm sorry for the lack of clarity of this field of physics, we're working on it). It’s also not clear what affirmation the sources 14,27,28,29 are supposed to support at l.84 because some of them support the existence of the physical phenomenon and some of them support the link between quantum mechanics and psychology.

**OK, we made the suggested change. The references address both temporal non-locality in purely physical and psycho-physical contexts and thus match the argument made one line above.
