# Supplementary material for: A preregistered multi-lab replication of Maier et al. (2014, Exp. 4) testing retroactive avoidance
Source: PLoS One. 2020 Aug 31;15(8):e0238373. doi: 10.1371/journal.pone.0238373 (PMC7458331; doi:10.1371/journal.pone.0238373)
Supplement: Attachment — Submitted filename: Response to Reviewers.docx [file pone.0238373.s004.docx]

Dear Dr. Dr. Naudet,

we would like to thank you for your additional comments on our revised manuscript entitled „A Preregistered Multi-Lab Replication of Maier et al. (2014, Exp. 4) Testing Retroactive Avoidance” submitted for publication to PLOS ONE. We tried to carefully respond to the comments you made concerning the abstract. In this document you will find our answers to your comments and a description of how we adjusted the text (see **).

Thank you and the reviewers again for the open and fair scientific discussion we had and your profound work on this paper.

Comment of the editor

I propose to use a structured abstract (i.e. more focused on the study methods and results).

** We shortened the theoretical part at the beginning and focused more exclusively on „retroactive avoidance“. We also described the design and the results in more detail.

Importantly, I ask you to delete any discussion about post-hoc analyses in the abstract.

**We deleted any discussion about the post-hoc findings.

I propose to add a few words in the abstract to highlight the main strengths and also limitations of the paper.

**We highlighted the strengths and limitations of the study at the end of the abstract.

Sincerely,

Markus Maier et al.
